# Mortality, greenhouse gas emissions and consumer cost impacts of combined diet and physical activity scenarios: a health impact assessment study

**DOI:** 10.1136/bmjopen-2016-014199

**Published:** 2017-02-21

**Authors:** Marko Tainio, Pablo Monsivais, Nicholas RV Jones, Christian Brand, James Woodcock

**Affiliations:** 1 MRC Epidemiology Unit, Centre for Diet and Activity Research, University of Cambridge School of Clinical Medicine, Institute of Metabolic Science, Cambridge, UK; 2 Systems Research Institute, Polish Academy of Sciences, Warsaw, Poland; 3 Transport Studies Unit, School of Geography and the Environment, University of Oxford, Oxford, UK

**Keywords:** Physical activity, Cycling, health inequality, NUTRITION & DIETETICS, greenhouse gas emissions, consumer costs

## Abstract

**Objective:**

To quantify changes in mortality, greenhouse gas (GHG) emissions and consumer costs for physical activity and diet scenarios.

**Design:**

For the physical activity scenarios, all car trips from <1 to <8 miles long were progressively replaced with cycling. For the diet scenarios, the study population was assumed to increase fruit and vegetable (F&V) consumption by 1–5 portions of F&V per day, or to eat at least 5 portions per day. Health effects were modelled with the comparative risk assessment method. Consumer costs were based on fuel cost savings and average costs of F&V, and GHG emissions to fuel usage and F&V production.

**Setting:**

Working age population for England.

**Participants:**

Data from the Health Survey for England, National Travel Survey and National Diet and Nutrition Survey.

**Primary outcomes measured:**

Changes in premature deaths, consumer costs and GHG emissions stratified by age, gender and socioeconomic status (SES).

**Results:**

Premature deaths were reduced by between 75 and 7648 cases per year for the physical activity scenarios, and 3255 and 6187 cases per year for the diet scenarios. Mortality reductions were greater among people of medium and high SES in the physical activity scenarios, whereas people with lower SES benefited more in the diet scenarios. Similarly, transport fuel costs fell more for people of high SES, whereas diet costs increased most for the lowest SES group. Net GHG emissions decreased by between 0.2 and 10.6 million tons of carbon dioxide equivalent (MtCO_2_e) per year for the physical activity scenarios and increased by between 1.3 and 6.3 MtCO_2_e/year for the diet scenarios.

**Conclusions:**

Increasing F&V consumption offers the potential for large health benefits and reduces health inequalities. Replacing short car trips with cycling offers the potential for net benefits for health, GHG emissions and consumer costs.

**Strengths and limitations of this study**

- We quantified the impact of five physical activity and six diet scenarios on all-cause mortality, greenhouse gas (GHG) emissions and consumer costs for the adult population of England, and stratified mortality and cost impacts by age, sex and socioeconomic status (SES).
- To the best of our knowledge, this is the first study that estimated health, consumer cost and GHG impacts of physical activity and diet scenarios, and estimated SES differences in the health and cost outcomes.
- The study was based on ‘what if’ hypothetical changes in physical activity and diet without consideration of how this change could be achieved in the study population.

## Introduction

Physical inactivity and low-quality diets are important risk factors for poor health at the global level. When expressing the health effects as disability-adjusted life-years, the Global Burden of Disease (GBD) 2013 study gave dietary risks as the most important and low physical activity as the 14th most important risk factors at the global level.^1^ Based on a specific analysis of the GBD 2013 data for England, dietary risks were the top risk factor, with low physical activity the ninth most significant factor.^2^ This indicates that changes in physical activity and diet can potentially have large impacts on public health. In this study, we investigated two potential changes that have been known to influence the above outcomes: (1) replacing short car trips with cycling^3–6^ and (2) increasing fruit and vegetable (F&V) consumption.^7^ ^8^

The benefits of such changes will vary in a population by age, gender and socioeconomic status (SES). The GBD 2013 analysis for England used a deprivation index varying from 1 (least deprived) to 5 (most deprived) and, in general, people in the most deprived group were more ill and had lower life expectancy than the average population.^2^ This suggests that the more deprived people may benefit more from increased physical activity and diet-related interventions than the less deprived.^9^ This raises questions on how to design interventions that target people on low SES,^10^ ^11^ and how the increase of cycling and F&V consumption would impact different SES groups.

Changes in cycling activity and F&V consumption may have other consequences, such as changes in individual's expenditure and greenhouse gas (GHG) emissions. If people substituted motorised transport with active transport (walking or cycling), the expenditure on transport and carbon emissions might decrease. The Living Costs and Food Survey for 2011 estimated that 14% of the household expenditure costs were due to transport, of which 55% were due to operational costs such as fuel expenditure.^12^ Similarly, GHG emissions from motorised travel may also decrease. In the UK, about 21% of domestic GHG emissions are from transport, with passenger cars accounting for 58.3% of total transport emissions.^13^ For passenger cars alone, trips under 5 miles account for ∼21% of GHG emissions,^13^ indicating that replacing short car trips with active transport may have significant GHG emission impacts.

In contrast, increased consumption of F&V could increase costs and GHG emissions. In the UK, 9.5% of domestic GHG emissions were from agriculture.^14^ The 2011 Living Costs and Food Survey estimated that 11% of total household expenditure was for food and non-alcoholic drinks, with 15% of this for F&V.^12^ Analysis of nationally representative data from the UK has shown that diets which meet the recommended intake of five portions of F&V per day are typically more expensive, and that F&V are more expensive than other food groups.^15^ ^16^ Similarly, data from the 2011 Living Costs and Food Survey showed that household expenditure for food and non-alcoholic drinks varied from 8% to 20% for the highest and lowest SES groups,^12^ respectively, indicating that changes in food costs may impact lower SES households more than others.

This study aimed to quantify the health, consumer cost and GHG emission changes for five physical activity and six diet scenarios for the adult population in England. The health benefits and changes in consumer costs were estimated by age, gender and SES of the study population, with further assessment of the changes in national GHG emissions. The main purpose was to quantify and compare how different SES groups would be affected under each scenario. To the best of our knowledge, this is the first study that examined the health, GHG emission and consumer cost impacts of physical activity and diet scenarios.

## Methods

### Overview and study population

**Figure 1:**
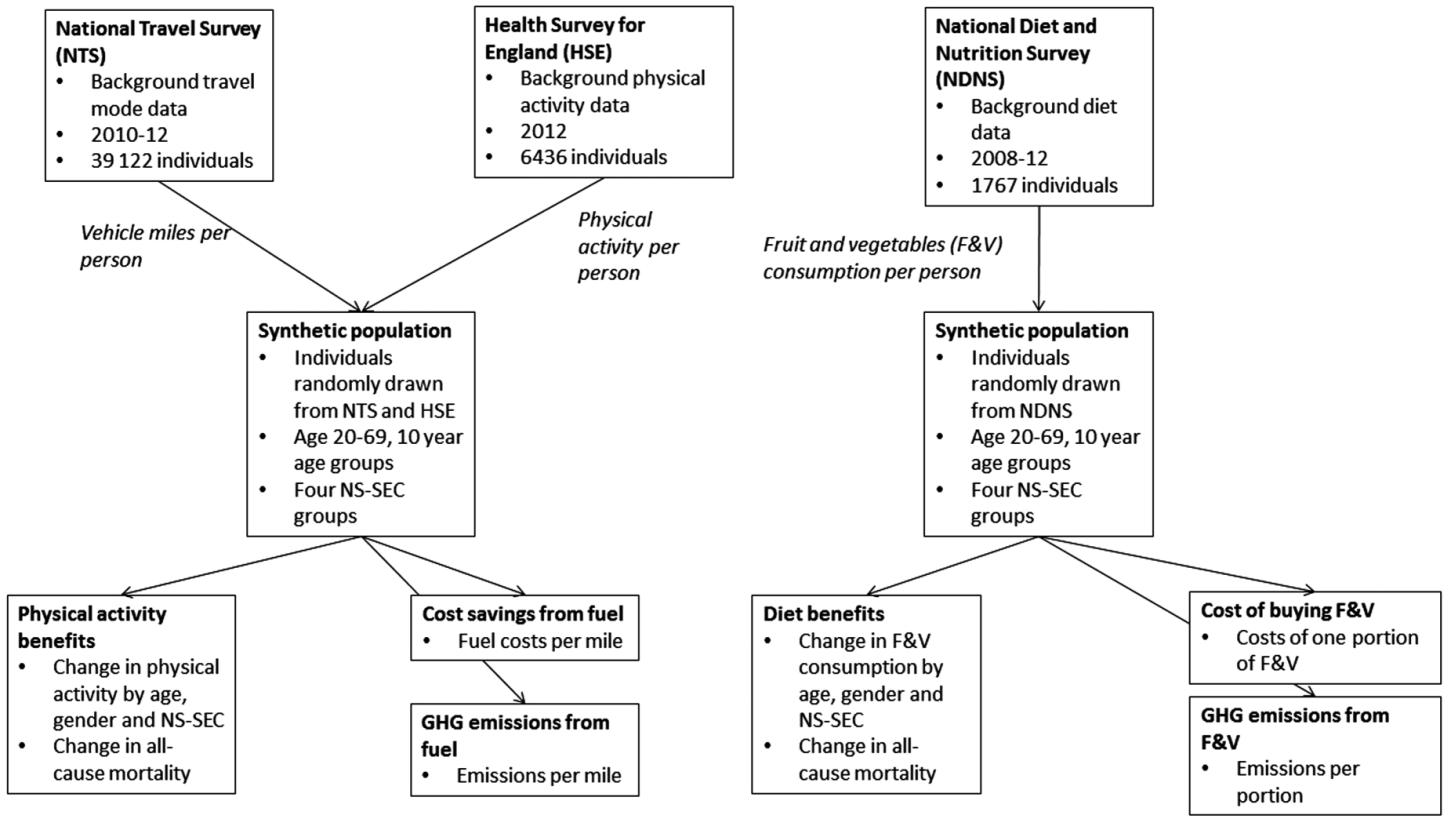
General overview of the calculation. NS-SEC, National Statistics Socio-Economic Classification.

We combined background travel, physical activity and F&V consumption data to generate a synthetic baseline population for England (figure 1). Health effects of physical activity and F&V consumption were modelled by combining changes in exposure with background all-cause mortality and dose–response functions (DRFs).

All calculations were performed on the working age population (20–69 years old) of England, totalling 34.2 million adults. The population was divided into five age groups (20–29, 30–39, 40–49, 50–59, 60–69), gender (male, female) and four SES groups (table 1).^17^ SES was measured by using the National Statistics Socio-Economic Classification (NS-SEC) system,^18^ which is an occupation-based classification of SES widely used in the UK. Specifically, we used three categories, the ordinal version of NS-SEC, with ‘not-classified’ as a fourth category. See online supplementary table S1 on how we aggregated NS-SEC classes from different surveys to the four categories used in this study.

**Table 1:** Percentage of population by age, gender and NS-SEC (total number of people 34.15 million)

	20–29	30–39	40–49	50–59	60–69	Sum
Female, NS-SEC						
Higher managerial, administrative and professional occupations	2.7%	4.2%	4.1%	3.1%	2.2%	16.3%
Intermediate occupations	2.1%	2.5%	3.2%	2.7%	2.5%	12.9%
Routine and manual occupations	3.1%	2.6%	3.3%	3.2%	3.4%	15.6%
Never worked and long-term unemployed	2.7%	1.1%	0.9%	0.5%	0.4%	5.6%
Sum	10.6%	10.4%	11.5%	9.5%	8.5%	50.4%
Male, NS-SEC						
Higher managerial, administrative and professional occupations	2.6%	4.3%	4.4%	3.4%	2.8%	17.6%
Intermediate occupations	1.7%	2.1%	2.6%	2.2%	2.0%	10.6%
Routine and manual occupations	3.7%	3.2%	3.6%	3.3%	3.2%	16.9%
Never worked and long-term unemployed	2.5%	0.7%	0.6%	0.4%	0.2%	4.5%
Sum	10.6%	10.3%	11.3%	9.3%	8.1%	49.6%

Data were obtained from the Office for National Statistics census 2011 database.^17^

NS-SEC, National Statistics Socio-Economic Classification.

10.1136/bmjopen-2016-014199.supp1 supplementary data

### Physical activity scenarios

#### Travel data

Background travel data for England were obtained from the National Travel Survey (NTS).^19^ The NTS is a rolling repeat cross-sectional annual household survey that records individual travel behaviour based on face-to-face interviews and a 7-day self-reported travel diary. Annual sample size is ∼16 000 individuals in 7000 households.

We obtained the year 2002–2012 NTS data from the UK data Service, from which years 2010–2012 data was extracted for this analysis.^19^ For each person (62 070), we extracted the age, gender, NS-SEC and travel mode data. We then allocated individuals to five age groups, two genders and four NS-SEC groups (see online supplementary table S1). We excluded individuals aged <20 and over 69 years, which gave a total of 39 122 individuals for the travel study sample.

#### Travel scenarios

For the physical activity scenarios, we replaced different shares of existing trips done by car with cycling, taking into account trip stage distances for trips involving travelling in a car (table 2). The NTS defines a trip as ‘a one-way course of travel having a single main purpose’. A trip can have multiple stages and new stages are defined when ‘there is a change in the form of transport or when there is a change of vehicle requiring a separate ticket’.^20^ The scenarios were created so that for each trip stage distance (<1, <2, <3, <5, <8 miles) the total miles per person were recorded and then summarised for each scenario. The respective scenarios were labelled as A—E, so A (<1 mile) to E (<8 miles; table 2). The car trip stages included any trips by ‘private car’, as defined in the NTS. Only stages where the person was the driver were taken into account.

**Table 2:** Description of scenarios and anticipated changes in health, GHG emissions and consumer costs

Name of the scenario	Description of scenario	Health	GHG emissions	Consumer cost
BAU	BAU. Background situation	0	0	0
A	All car stages 1 mile or shorter are replaced with cycling	+	+	+
B	All car stages 2 miles or less long are replaced with cycling	+	+	+
C	All car stages 3 miles or less long are replaced with cycling	+	+	+
D	All car stages 5 miles or less long are replaced with cycling	+	+	+
E	All car stages 8 miles or less long are replaced with cycling	+	+	+
F	All people eat 1 portion of fruit and vegetables more per day	+	**−**	**−**
G	All people eat 2 portions of fruit and vegetables more per day	+	**−**	**−**
H	All people eat 3 portions of fruit and vegetables more per day	+	**−**	**−**
I	All people eat 4 portions of fruit and vegetables more per day	+	**−**	**−**
J	All people eat 5 portions of fruit and vegetables more per day	+	**−**	**−**
K	All people eat at least 5 portions of fruit and vegetables per day	+	**−**	**−**

Plus (+) means positive consequences due to scenario (eg, less cost for individuals, less GHG emission, better health) and minus (**−**) negative consequences.

BAU, business as usual; GHG, greenhouse gas.

For cycling we obtained duration and distance of each stage done by bike, from which we calculated average cycling speed by age and gender (see online supplementary figure S1). On the basis of this, we calculated increases in cycling time by dividing the miles travelled by car (from A to E above) by the average cycling speed. We could therefore estimate the increase in cycling time (hours) per week, if all stages of a particular trip stage length travelled by car were to be substituted by cycling.

#### Background physical activity data

The DRF for physical activity is non-linear.^21–23^ To estimate health benefits of increased cycling, we needed to take into account background physical activity of the target population. Background physical activity data were obtained from the 2012 Health Survey for England (HSE)^24^—an annual survey that monitors the health of the English population. The year 2012 data were obtained because it is the latest HSE survey with a physical activity focus.

The HSE provided us with activity data on ‘walking’ and ‘doing sport’ by age, gender, NS-SEC and duration per week for a total of 6436 individuals aged 20–69 years. For each individual, we calculated background physical activity levels by converting ‘time walking’ and ‘doing sport’ to metabolic equivalent of task (METs) hours. METs are a measure of intensity of physical activity that can be used to aggregate different activities to one physical activity unit.^25^ One MET refers to the energy cost of being seated (resting). Total MET hours per week can be calculated by multiplying the time spent on different physical activities with the average MET of that activity. In this study, we used marginal METs (mMETs) which refers to energy use above rest. mMETs are calculated by subtracting 1 from the MET rate of different activities and hence give greater relative weight to more intensive activity.

From the HSE we also obtained activity time for walking and 10 different sports (see online supplementary table S2). Average mMETs for walking and each different sport were selected from the Compendium of Physical Activities 2011 database^25^ by comparing the definition of the activities in HSE 2012 to the description of activities in the Compendium. To account for uncertainty in the estimates, we assumed a ±25% uncertainty range around the average mMETs for each activity.

#### Integration of travel and health data

Individuals from the NTS and HSE data sets were integrated randomly to create a synthetic population for further analysis (figure 1). The integration was done so that a random person in the same age group (5), gender (2) and SES (4) was drawn separately from the NTS and the HSE and then matched together to create a synthetic person with a background travel pattern based on the NTS and a background non-travel physical activity based on the HSE. For each age, gender and socioeconomic group, 1000 random individuals were drawn from the NTS and the HSE. All further calculations were based on this synthetic population. Comparison of the synthetic population with the original survey data showed that average input values had about ±5% differences between the synthetic population and the survey data samples for most age, gender and NS-SEC groups (see online supplementary table S3).

#### Fuel cost and GHG emissions

Fuel consumption savings as a result of substituting car travel with cycling were estimated based on the tailpipe (exhaust) carbon dioxide (CO_2_) emissions factors for petrol and diesel cars in the UK (see online supplementary table S4). Values for CO_2_ were obtained from the Greenhouse Gas Conversion Factor Repository^26^ and the average CO_2_ content per fuel from UK Energy Statistics.^27^ By combining this information with the average fuel price^28^ and with the proportion of car fleet between petrol and diesel cars,^29^ we estimated that the average fuel cost 0.19£/mile (at 2015 prices; see online supplementary table S3). The Automobile Association of the UK estimated that the running costs of petrol and diesel cars vary from 0.19 to 0.29£/mile and 0.17 to 0.28£/mile, respectively. Therefore, our estimate for fuel costs can be considered a conservative estimate of the running costs of a car. Given the wide distribution of tailpipe CO_2_ emissions from cars and the variability in fuel costs across time and space, we assumed a ±50% uncertainty around these average estimates.

Similar to the above, changes in GHG emissions for the different scenarios were calculated based on the Greenhouse Gas Conversion Factor Repository.^26^ The GHG emission data were based on carbon dioxide equivalent (CO_2_e) emissions factors that take into account all transport-related GHG emissions. The average CO_2_e emission factor for cars was assumed to be 0.32 kg CO_2_e/mile. We assumed a ±25% uncertainty for this emission factor in our sensitivity analyses. National CO_2_e emission reduction was calculated by estimating the average reduction of car driving per person in different scenarios and then multiplying this with the study population by age, sex and NS-SEC (table 1).

### Diet scenarios

#### Background F&V data

Background F&V data were obtained from the rolling programme of the National Diet and Nutrition Survey (NDNS).^30^ The NDNS collects nationally representative data on food consumed by individuals and covers all four countries in the UK. We used all available data for adults aged between 20 and 69 years (n=1 767). From the NDNS we extracted the background F&V consumption per person, stratified by age, gender and NS-SEC. We followed the NDNS approach in counting a single portion of F&V as 80 g of consumed mass.

#### F&V scenarios

The first five diet scenarios (F–J) assumed that everyone in the study population would consume 1, 2, 3, 4 or 5 portions of F&V *more* per day, respectively (table 2). In scenario K, we assumed that everyone who does not consume five portions of F&V per day would increase their consumption to five portions per day. This represents a scenario where everyone in the study population would eat five portions a day or more. The remainder of the diet was assumed to stay unchanged, therefore implying that any additional F&V intake was not replacing other food items.

#### F&V costs and GHG emissions

One portion of F&V was assumed to cost £0.22, based on Jones and Monsivais.^31^ In that study, the average costs of F&V were predicted by combining food price information with the food consumption information from NDNS. Thus, the costs of F&V reflect the average costs of F&V when taking into account how much and which F&Vs are eaten in the UK. To account for uncertainty in portion cost, we assumed a ±25% sensitivity range around the average cost of one F&V portion.

The embedded GHG emissions of F&V were assumed to be 0.086 kg CO_2_e/portion (for fruit) and 0.166 kg CO_2_e/portion (for vegetables). Emissions were calculated by combining the food item-specific GHG emissions from Audsley *et al*^32^ with the dietary intake information from the European Prospective Investigation into Cancer and Nutrition—Norfolk, UK cohort.^33^ A similar method was used by Briggs *et al*^34^ and Scarborough *et al*.^35^ The change in national CO_2_e emissions was estimated by first calculating the per person increase of portions by age, sex and NS-SEC, and then multiplying the average CO_2_e increase per person with the background population (table 1).

#### DRFs and background mortality

Changes in all-cause mortality were estimated for physical activity and F&V consumption. For physical activity we obtained DRFs from a systematic review and meta-analysis by Woodcock *et al*.^21^ We used relative risk (RR) values of 0.81 (95% CI 0.77 to 0.85) comparing 8.6 mMEThours/week against no activity. Woodcock *et al*^21^ reported several different shapes of DRFs varying from nearly linear to non-linear ones. We assumed that the shape of the DRF is uncertain and varied by power log between 0.25 (non-linear) and 1.00 (linear), with 0.5 as a central estimate.

For F&V consumption we adopted DRFs from the dose–response meta-analysis by Wang *et al*^36^ who pooled results from seven epidemiological cohort studies that had examined associations between F&V consumption and all-cause mortality. They estimated both linear and non-linear DRF for F&V consumption. In this study, we used the non-linear DRF. Compared with people who eat zero portions of F&V per day, the estimated HRs for all-cause mortality were 0.92, 0.85, 0.79, 0.76, 0.74 and 0.74 for one, two, three, four, five and six or more portions of F&V per day, respectively. We used these HRs to estimate the mortality change after increased F&V consumption. Thus, we assumed that after the sixth portion of F&V per day there would be no further benefit.

The contribution of physical activity and F&V to all-cause mortality was calculated by applying the following equation:
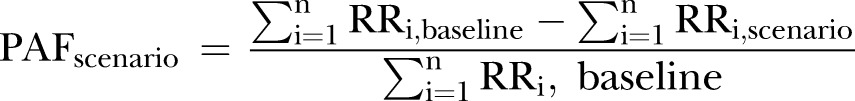


Where PAF is the population attributable fraction, RR_i,baseline_ is the RR at the exposure level i in baseline scenario, RR_i,scenario_ is the RR at the exposure level i in scenario and n is the number of people affected by the scenario.

The change in all-cause mortality by age, sex and NS-SEC was calculated by multiplying the PAFs with the background mortality. Background mortality for two calendar years for the population of England and Wales was obtained from the ad hoc database of the Office for National Statistics.^37^ The number of deaths per year for the study population was estimated from these data by adjusting the number of deaths for England and Wales with English mortality counts for the year 2011,^38^ and by halving the values to calculate mortality counts for 1 year. Adjusted mortality data for the study population are presented in table 3.

**Table 3:** Percentage of deaths by age, sex and NS-SEC for England (total number of deaths 103 843 cases per year)

	20–29	30–39	40–49	50–59	60–69	Sum
Female						
Higher managerial, administrative and professional occupations	0.2%	0.5%	1.6%	3.5%	6.9%	12.6%
Intermediate occupations	0.1%	0.3%	0.9%	2.0%	5.0%	8.2%
Routine and manual occupations	0.2%	0.4%	1.4%	3.2%	8.1%	13.4%
*Never worked and long-term unemployed	0.4%	0.6%	1.2%	1.6%	1.8%	5.6%
Sum	0.9%	1.9%	5.0%	10.2%	21.8%	39.7%
Male						
Higher managerial, administrative and professional occupations	0.2%	0.6%	1.6%	3.6%	7.8%	13.8%
Intermediate occupations	0.2%	0.5%	1.2%	2.8%	6.8%	11.4%
Routine and manual occupations	0.7%	1.3%	3.1%	6.8%	15.8%	27.8%
*Never worked and long-term unemployed	0.8%	1.0%	1.7%	1.9%	1.8%	7.2%
Sum	2.0%	3.4%	7.7%	15.0%	32.2%	60.3%

Data were obtained from the Office for National Statistics ad hoc data service for England and Wales, and were adjusted for the population of England with the mortality numbers per age and gender^37^.

NS-SEC, National Statistics Socio-Economic Classification.

#### Implementation of the model

The model was implemented using the Monte Caro simulation program Analytica V.4.6 (http://www.lumina.com) running 1000 iterations. Monte Carlo simulation was used to predict 95% credible intervals for each uncertain output variable.

## Results

**Figure 2:**
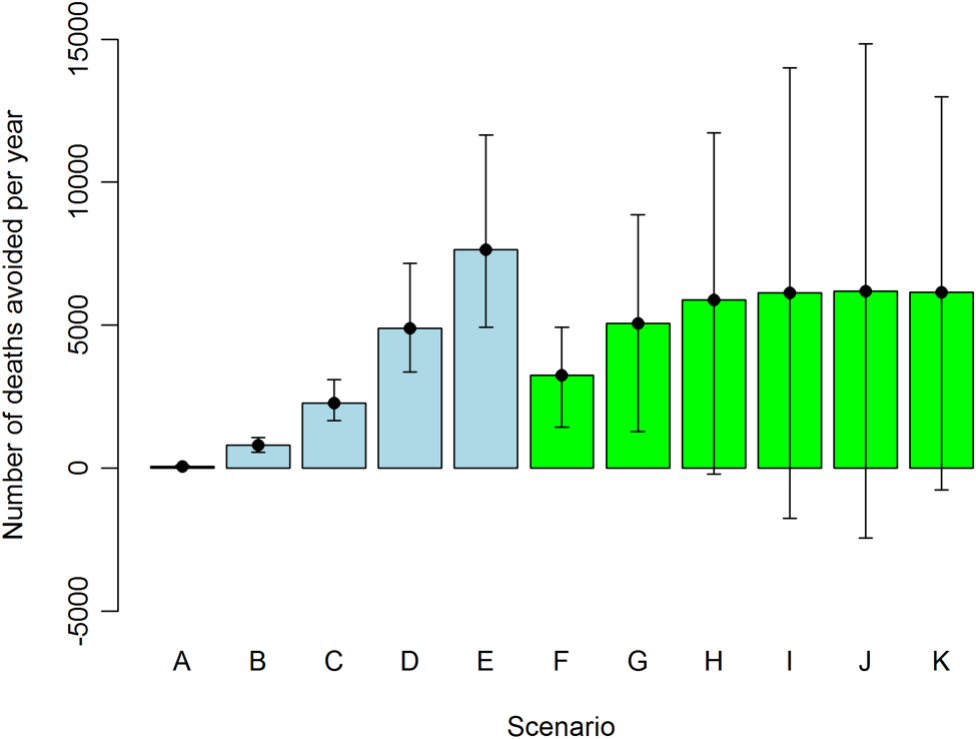
Avoidable deaths per year (mean and 95% credible interval) in England for different scenarios. See table 2 for description of scenarios and online supplementary table S4 for numerical values.

The health benefits for the different scenarios varied from 75 (95% credible interval 47 to 113) deaths avoided per year (scenario A) to 6187 (95% CI −2430 to 14 836) deaths avoided per year (scenario J; figure 2, see online supplementary table S5 in supplementary material for the numerical values). In all scenarios, the number of deaths averted was higher for men. In physical activity scenarios, women increased cycling more than men (see online supplementary figure S2) while the background mortality was higher for men (table 3). When combined, the overall premature deaths averted was slightly higher for men. For the diet scenarios (F–J), both genders increased F&V consumption by one or more portions per day and this led to a higher mortality decrease for men than for women (figure 2). Also, in scenario K, where the portion increase depended on background F&V consumption, men benefited more than women.

About 73% of people increased F&V consumption in scenario K. The percentage of population changing their physical activity was 13%, 33%, 43%, 51% and 55% for scenarios A–E, respectively. The mean increase in cycling time among those changing their behaviour ranged from 0.2 to 5.0 hours/week (see online supplementary figure S2). This had a noticeable impact on population physical activity. In the baseline case, 44% of the study population were engaged in at least 150 min of physical activity per week with medium intensity (8.75 mMEThours/week); in scenario E, this increased to 67% (see online supplementary figure S3). About 30% of the population were achieving 300 min of medium intensity physical activity at baseline; in scenario E, this increased to 52% (see online supplementary figure S3).

### Estimated health impact of scenarios by SES

Online supplementary figure S4 shows the percentage changes in all-cause mortality for different scenarios and socioeconomic groups, with deaths avoided shown in online supplementary figure S5. For the physical activity scenarios (A–E), the percentage decrease in mortality was similar across the NS-SEC groups for scenarios A–C, but in scenarios D and E the lowest NS-SEC group (group 3, routine and manual occupations) showed smaller benefits than the rest. In all physical activity scenarios, people outside the NS-SEC classification showed smaller benefits than people within the classification. The lowest NS-SEC group and people outside the NS-SEC classification benefited most in the diet scenarios (F–K). Importantly, over half of the avoidable deaths would occur in the lowest NS-SEC group for all diet scenarios (group 3, routine and manual occupations; see online supplementary figure S5).

### Costs

**Figure 3:**
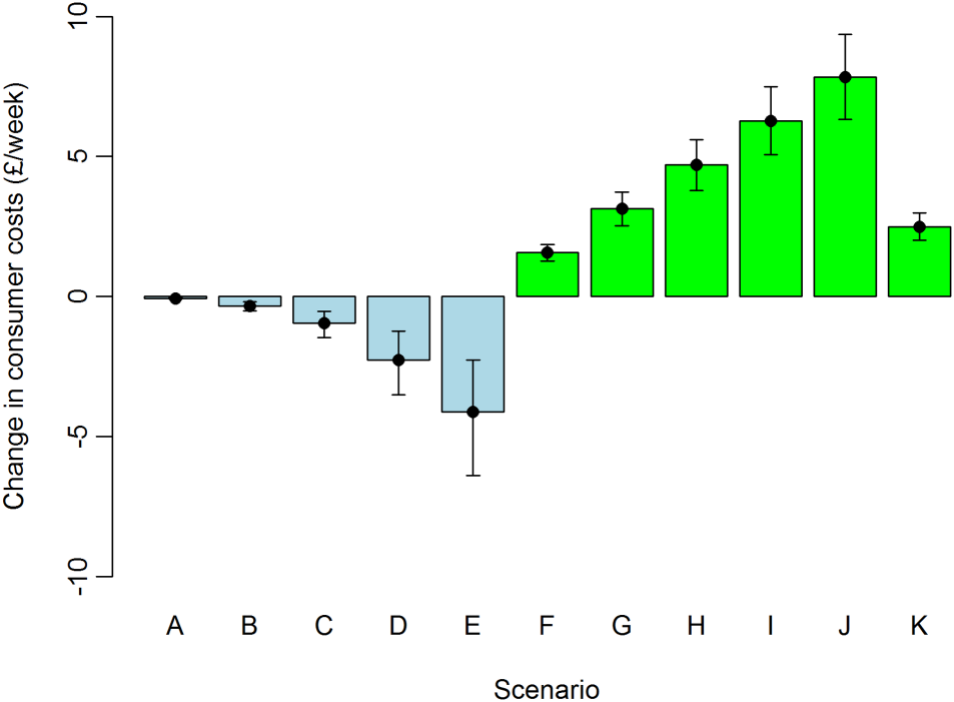
Change in consumer costs in different scenarios (mean and 95% credible interval).

Figure 3 provides changes in weekly average consumer costs per person for scenarios A–K, showing a range of positive and negative outcomes. Scenario E, for instance, was estimated to decrease per person costs of the study population by £4.12 (95% CI £2.26 to £6.38) per person per week; and scenario J was estimated to increase costs by £7.84 (95% CI £6.32 to £9.36) per person per week (see online supplementary table S5). These represent 15% and 34% changes in mean transport (£27.38) and food and non-alcoholic drinks (£22.83) costs per week, respectively^12^ (see online supplementary table S6). For the physical activity scenarios (A–E), fuel cost savings were highest among the highest NS-SEC group, with changes proportionally similar between the different NS-SEC groups. For diet scenario K, the cost increases were highest for the lowest NS-SEC group (£2.98 per person per week) and lowest for the highest NS-SEC group (£1.99 per person per week). For scenario J, the lowest and highest NS-SEC groups increased food and alcoholic drinks costs by 39% and 23%, respectively, indicating significant socioeconomic differences in the proportional costs between scenarios.

### GHG emissions

**Figure 4:**
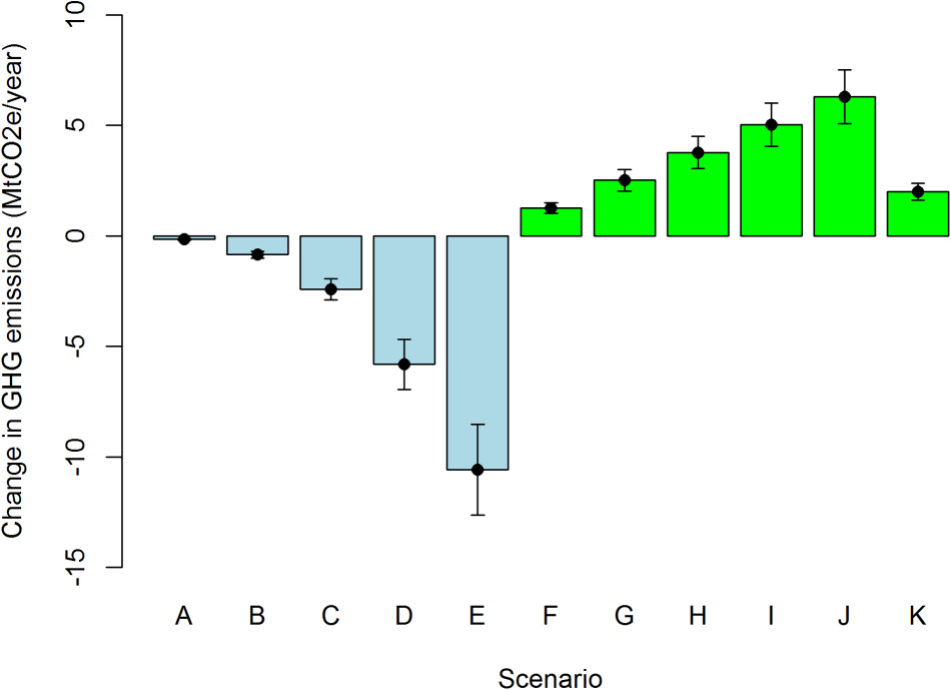
Change in GHG emissions (MtCO_2_e/year) in different scenarios (mean and 95% credible interval). GHG, greenhouse gas; MtCO_2_e, million tons of carbon dioxide equivalent.

Changes in GHG emissions in different scenarios are presented in figure 4. The CO_2_e emissions reductions due to decreased car use were 67 times higher in scenario E when compared with scenario A. Owing to increased consumption of F&V, scenario J gave five times higher CO_2_e emissions than scenario F. When compared with annual CO_2_e emissions from passenger cars in England (56.5 MtCO_2_e/year, in 2012^39^), total emissions from cars would drop by between 0.3% (scenario A) and 19% (scenario E). The diet scenarios were equivalent to an increase in GHG emissions from agriculture in England (which were 28.27 MtCO_2_e/year in 2012^39^) of between 4.4% (scenario F) and 22% (scenario J). In comparison, scenario K showed an increase in GHG emissions of 7.1%.

## Discussion

This study simultaneously estimated the health effects, consumer costs and GHG emission changes of several physical activity and diet scenarios for the working age population of England. While the direction of the changes was perhaps as expected, this study allowed us, for the first time, to quantify the magnitude of the impacts across different outcomes and population groups. Replacing short car trips with cycling and increasing consumption of F&V would create large health benefits for the population. While replacing short car trips with cycling would benefit more people with high SES, increasing consumption of F&V would benefit more people with lower SES. Both would have significant impacts on consumer costs so that diet scenarios would increase food and non-alcoholic drinks costs for people in low SES groups, whereas for the physical activity scenarios cost differences between SES groups are smaller. Both physical activity and diet scenarios implied significant changes to transport and agricultural GHG emissions in England.

### Meaning of the study: possible explanations and implications for clinicians and policymakers

Increasing physical activity (scenarios A–E) and F&V consumption (scenarios F–K) would have large health benefits in their own right. What this study added is quantifying and comparing those benefits against each other. Even relatively small changes in travel behaviour, such as replacing the shortest (<1 mile long) car trips with cycling (scenario A), would have modest health benefits, with 75 premature deaths avoided per year (figure 2). The diet scenarios showed even greater potential for health benefits, with ∼3000 deaths avoided per year if people would eat one portion of F&V more per day (scenario F, figure 2). By quantifying benefits of such scenarios, this study provides information that can be used in cost-benefit studies that assess health and diet interventions.

We also quantified the health and cost changes between socioeconomic groups. In physical activity scenarios (A–E), population health was improved most for the people in the highest SES group (see online supplementary figures S4 and S5). Since more people of higher SES make short car trips, more people benefit from the shift to active modes (see online supplementary figure S6). Thus, replacing car trips with cycling would improve health overall but would not reduce health inequalities. It is important to note that we have not modelled an increase in cycling per se but a shift from cars to bikes. The results might have been different if we would have considered a potential shift from buses to cycling, or the potential for new cycling trips for those who do not have access to a car, for example, if cycling helped access new employment opportunities.

The diet scenarios showed great potential for reducing health inequalities by benefiting most people in low SES (see online supplementary figures S4 and S5). In all diet scenarios (F–K), approximately half of all the deaths avoided were in the lowest SES group. However, people in the lowest SES group would have a significant percentage increase in their food and non-alcoholic drinks costs (see online supplementary table S6). This suggests that affordability or compensation needs to be considered for interventions aiming to increase F&V consumption.

The story was more straightforward for changes in GHG emissions (figure 4), with physical activity scenarios decreasing transport-related GHG emissions and diet scenarios increasing emissions by several percentage points over baseline GHG emissions from transport and agriculture. This has obvious implications for designing integrated health and climate change mitigation policies, further contributing to the debate about how to achieve ‘co-benefits’ across outcomes and policy objectives.

### Strengths and limitations

The strength of our study is the combination of health, consumer cost and GHG emission effects of physical activity and diet scenarios to one assessment. We could show health and consumer cost effects by age, gender and SES to indicate which scenarios would most likely reduce health inequalities, and what would be the likely magnitude of the impact. To the best of our knowledge, this is the first study that has integrated such a variety of pathways (physical activity, diet) to outcomes (health, cost, GHG emissions).

This study took advantage of the good-quality survey data routinely collected in England from health, transport and diet (figure 1). On the basis of these data, we could create individual-based scenarios where the changes in physical activity and diet are calculated on an individual level, rather than based on population averages. For physical activity and F&V consumption, the DRF for all-cause mortality is non-linear and assessing the changes in health on an individual level allows us to better take into account these non-linearities. Also, by using individual-level data, we could estimate costs per person, rather than average changes.

To combine and quantify all the outcomes, we needed to make some simplifications, with potential impacts to results.

First, for the diet scenarios, we only took into account increase in F&V consumption independent of the possible other changes in diet. If increases in F&V consumption are not compensated for by reductions in other areas, body mass index (BMI) would increase, leading to negative health effects. If there were compensatory energy intake reductions in the consumption of other food groups, this could have positive or negative changes in health, depending on what food items are compensated. For example, a shift from meat consumption to F&V could reduce GHG emissions and consumer costs, while a reduction in sugar consumption would benefit health but have little effect on GHG emissions or cost.^33^

To predict these changes, some modelling studies, such as Nnoaham *et al*,^40^ have predicted changes in multiple food groups. In that study, one of the scenarios (scenario 4) predicted an ∼10% increase in F&V consumption with 1% or less changes in calories, saturated fat and salt intake. The health benefits of this scenario were 3700 deaths avoided with only minor negative health effects related to obesity. This indicates that the health effects were mainly due to changes in F&V consumption and not due to BMI.

Second, we based our background travel data on stages of the trips. This means that we did not take into account journeys that consisted of several stages (eg, driving and walking). This will add uncertainty to our results since not all the stages can be cycled if they are part of a multistage journey. However, since our purpose was to estimate creditable upper bound health benefits of different scenarios, rather than effects that can be achieved, we consider this simplification justifiable.

Third, for the physical activity scenarios, we took into account only the benefits of physical activity without consideration of risks. Several other health impact assessment studies have examined health effects of mode shift from motorised transport to active transport (walking, cycling) in the UK and elsewhere by taking into account physical activity, air pollution and injury risks.^4^ ^6^ The general conclusion from these studies has been that physical activity benefits are larger than the risks posed by air pollution and injuries.^41^ ^42^ A recent study that compared physical activity benefits with air pollution risks has confirmed that with the air pollution levels in England, individual benefits from physical activity will be substantially higher than harms.^43^ Thus, we assumed that our results would be similar even if the injury and air pollution risks would be taken into account.

Fourth, the diet scenarios result in changes to all people consuming fewer than six and, in one case, five portions of F&V per day, while the physical activity scenarios affect only those doing car trips shorter than 1–8 miles. If we had devised a physical activity scenario that affected all those doing less than the recommended levels, then the benefits would have been larger. However, replacing short car trips with cycling is often cited as a mechanism for increasing population levels of physical activity and provides a more interesting comparison than a more abstract scenario. Cycling could also come from other modes than driving. If cycling replaces other motorised modes, then physical activity benefits would be greater and spread across a wider demographic, providing more equitable benefits. On the other hand, if cycling would replace walking, then physical activity and associated benefits would be smaller.

Fifth, in diet scenarios, we used average CO_2_e emission factors based on the food items currently consumed in England. However, there exists wide variation in average CO_2_e emissions per different food items and the actual increase of the emissions would vary a lot between different food items. For example, by following Audsley *et al*, the emission factor for cabbages produced in the UK would be 0.0176 kg CO_2_e/portion (five times smaller than the emission factor used in our study). On the other scale are beans produced outside Europe, with an emission factor of 0.816 kg CO_2_e/portion (6.5 times higher than the emission factor used in this study). Thus, the environmental impact could be improved by focusing on increasing uptake of lower emission F&Vs.

### Comparison to other studies, discussing important differences in results

We modelled the health effects directly from physical activity and F&V consumption to health outcomes without intermediate risk factors. For example Cecchini *et al*^44^ estimated health effects of physical activity and diet scenarios through one intermediate risk factor (BMI), and through three proximal risk factors (blood pressure, cholesterol, glycaemia). The PRIME (Preventable Risk Integrated ModE) model^45^ estimates physical activity and diet risks both directly and through BMI. On the basis of the comparison of our results to the apple per day scenario,^7^ calculated with the PRIME model and described in greater detail in online supplementary material, our estimates are of a similar magnitude but smaller, providing some cross-model validation of the results.

The results of physical activity scenarios (A–E) are similar to those made in a previous study for Barcelona, Spain,^13^ but much smaller than similar study in New Zealand^14^ (see online supplementary material for details). Both of these studies adopted the linear DRFs from the WHO Health Economic Assessment Tool (HEAT) to estimate health benefits of physical activity.^46^ Earlier studies have found that linear DRFs from HEAT suggest larger health benefits than the non-linear functions applied in our study, but this finding is likely to be scenario specific.^47^

Diet scenario (F–K) results are smaller than the two UK modelling studies that have examined health benefits of eating more apples^7^ or F&V^8^ (see online supplementary material for details). Both of these studies included an older population than did our study and this might explain the smaller health benefits in our study. In our study, diet scenarios had also significant SES differences in proportional costs indicating that people in low SES might have financial difficulties to increase F&V consumption. A recent review of dietary interventions concluded that price interventions appear to decrease inequalities.^10^

A previous UK study estimated CO_2_ emissions of transport by purpose and the distance of the trips.^13^ In that study, 21% of the transport-related CO_2_ emissions were from trips <5 miles long.^13^ In our study, scenario D was assumed to reduce CO_2_e emissions by 5.8MtCO_2_e/year (figure 4), which represents 10.3% of total cars-related and taxis-related CO_2_e emissions in England in 2012. A number of earlier studies have also observed highly unequal distributions of CO_2_ emissions from motorised travel,^48–50^ with the top fifth of the population generally producing more than three-fifths of the emissions. In our study, the CO_2_e emission reductions were highest among the highest NS-SEC groups and lowest in the ‘never worked group’ indicating SES differences in the GHG emissions.

For diet, previous UK modelling study combining health and climate effects concluded that adopting diets with low GHG emissions would provide large health co-benefits through changes in fruit, vegetable, red meat and processed meat consumption.^51^ That study optimised GHG emission reductions by taking into account potential acceptance of the new diet and dietary recommendations from the WHO, and estimate health as co-benefit following the climate friendlier diet. This shows that an increase in F&V consumption can be part of a decrease in overall GHG emissions from food production if other parts of the diet change sufficiently.

### Unanswered questions and future research

Our scenarios assumed hypothetical change in the physical activity and diet behaviour without consideration of how such changes could be achieved in a population. The next challenge for impact assessment research is to predict how individual actions or programmes would change the behaviour of the population, and what consequences these changes would have. For example, a large body of evidence indicates that providing safe, comfortable and direct routes for cycling to popular destinations is the most effective method for achieving mass cycling. This evidence is based on the experience of high cycling countries,^52^ ^53^ stated preferences on infrastructure and fear of motor traffic,^54^ studies on the injury risk reductions on protected infrastructure,^55^ and is beginning to be supported by evidence from natural experiment studies.^56^

For diet, the econometric analysis of the UK 5 a day marketing campaign, aimed at increasing F&V consumption in the population, concluded that the campaign increased average F&V consumption by 0.3 portions per day.^57^ A meta-analysis of randomised controlled trials in retirement age adults increased F&V intake by approximately one portion per day both the short term (less than a year) and long term (more than a year).^58^ Translating this kind of evidence for impact assessment is challenging, but required, to estimate the likely effectiveness of different interventions.

## Conclusions

We estimated health benefits of physical activity (mode shift from car to bicycle) and diet (eating more F&V) related scenarios for the working age population of England. We also estimate changes in GHG emissions and costs. We found out that both replacing short car trips with cycling and increasing consumption of F&V would have large health benefits for the population. Physical activity scenarios benefited most people with high NS-SEC status, and diet scenarios people with low NS-SEC. Since the physical activity scenarios lowered costs and the diet ones increased them, this means that costs were increased most for people of lower SES and reduced most for people of high SES. These results give quantitative information on potential health, GHG emission and consumer cost changes that physical activity-related transport scenario and F&V-related diet scenarios could achieve at the population level.
