# Supplementary material for: Mortality, greenhouse gas emissions and consumer cost impacts of combined diet and physical activity scenarios: a health impact assessment study
Source: BMJ Open. 2017 Feb 21;7(2):e014199. doi: 10.1136/bmjopen-2016-014199 (PMC5337665; doi:10.1136/bmjopen-2016-014199)
Supplement: supplementary data [file bmjopen-2016-014199supp.pdf]

Supplement material to Tainio et al. Mortality, greenhouse gas emissions, and consumer cost impacts of combined diet and physical activity scenarios: A Health Impact Assessment study. BMJ Open 2016.

Table S1: Three classes NS-SEC codes used in this study (top-row). Rows show the aggregation of NS-SEC classes from different surveys to three class version used in this study. As a fourth class we had “Never worked and long-term unemployed” to cover all population groups. See (1) for details of NS-SEC.

	**1. Higher managerial, administrative and professional occupations**	**2. Intermediate occupations**	**3. Routine and manual occupations**	**Never worked and long-term unemployed**
**National Travel Survey (NTS)**	• Managerial and professional occupations	• Intermediate occupations and small employers	• Routine and manual occupations	• Never worked and long-term unemployed<br>• Not classified (including students)<br>• DNA
**Health Survey for England (HSE)**	• Higher managerial and professional occupations<br>• Lower managerial and professional occupations	• Intermediate occupations<br>• Small employers and own account workers	• Lower supervisory and technical occupations<br>• Semi-routine occupations<br>• Routine occupations	• Never worked and long term unemployed
**National Diet and Nutrition Survey (NDNS)**	• Higher managerial and professional occupations<br>• Lower managerial and professional occupations	• Intermediate occupations<br>• Small employers and own account workers	• Semi-routine occupations<br>• Lower supervisory and technical occupations<br>• Routine occupations	• Never worked<br>• Other<br>• Item not applicable<br>• Don't know
**Mortality data**	• Higher Managerial and Professional Occupations<br>• Lower Managerial and Professional Occupations	• Intermediate Occupations<br>• Small Employers and Own Account Workers	• Lower Supervisory and Technical Occupations<br>• Semi-routine Occupations<br>• Routine Occupations	• Not Classified

Figure S1: Mean cycling speed by age and gender. Based on NTS 2010-12 (2).

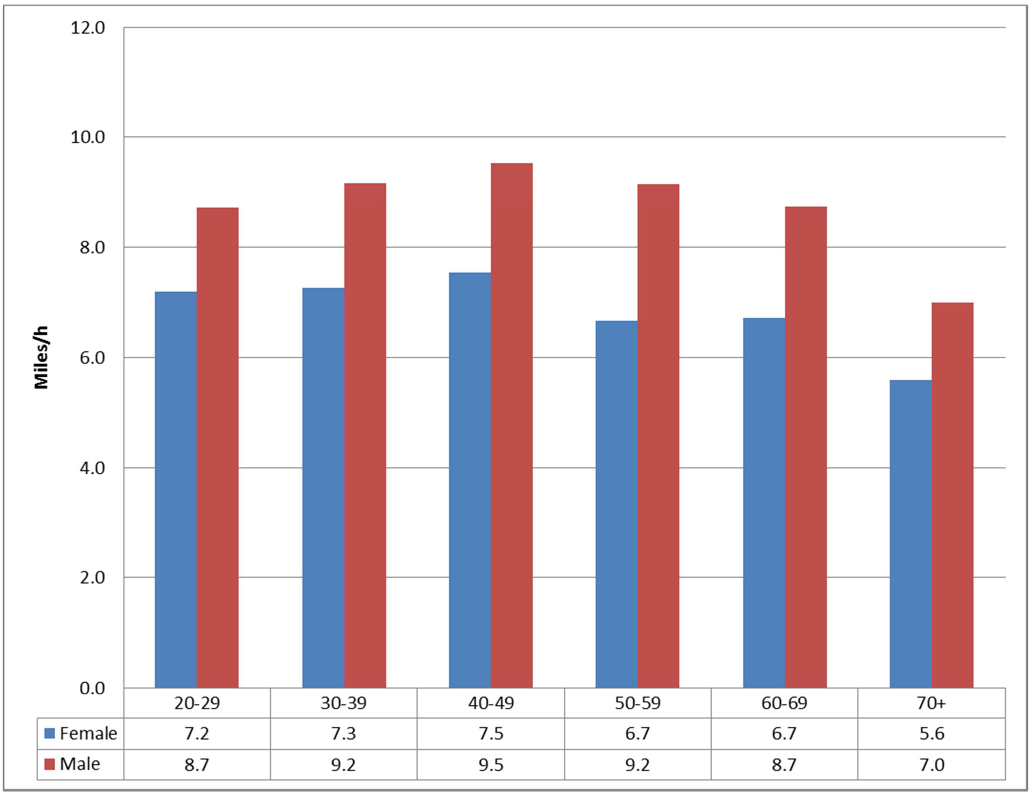

Table S2: Mean marginal METs (mMETs) for walking and different sports. Marginal METs were defined by comparing the MET values from Compendium of Physical Activities (3) with the description of activities in HSE survey (4). For uncertainty we assumed +-25% uncertainty around the average mMETs.

**Activity**	**Average mMET**
Walking	3.9
Swimming	6.0
Cycling	6.8
Working out	6.0
Aerobics/gymnastic	7.3
Dancing	5.0
Running	8.4
Football	7.65
Badminton/tennis	7.0
Squash	7.3
Exercises	8.0
Any other sport	6.0

Table S3: Validation of synthetic population with the background data. The percentage shows how much the synthetic mMETs (A), miles driven less for scenario C (B) and average portions of F&V (C) differ between synthetic population and data.

A

**Female**	**20-29**	**30-39**	**40-49**	**50-59**	**60-69**
1. Higher managerial, administrative and professional occupations	99%	103%	95%	93%	108%
2. Intermediate occupations	99%	107%	106%	110%	104%
3. Routine and manual occupations	97%	112%	93%	102%	97%
*Never worked and long-term unemployed	94%	98%	100%	106%	108%
**Male**	**20-29**	**30-39**	**40-49**	**50-59**	**60-69**
1. Higher managerial, administrative and professional occupations	99%	93%	106%	100%	100%
2. Intermediate occupations	105%	98%	104%	115%	101%
3. Routine and manual occupations	97%	95%	105%	102%	92%
*Never worked and long-term unemployed	104%	98%	100%	91%	99%

B

**Female**	**20-29**	**30-39**	**40-49**	**50-59**	**60-69**
1. Higher managerial, administrative and professional occupations	93%	101%	100%	102%	103%
2. Intermediate occupations	104%	102%	95%	95%	101%
3. Routine and manual occupations	97%	99%	99%	103%	100%
*Never worked and long-term unemployed	104%	104%	99%	94%	100%
**Male**	**20-29**	**30-39**	**40-49**	**50-59**	**60-69**
1. Higher managerial, administrative and professional occupations	96%	103%	106%	96%	96%
2. Intermediate occupations	100%	92%	104%	100%	101%
3. Routine and manual occupations	106%	100%	95%	103%	98%
*Never worked and long-term unemployed	97%	99%	102%	103%	105%

C

**Female**	**20-29**	**30-39**	**40-49**	**50-59**	**60-69**
1. Higher managerial, administrative and professional occupations	97%	107%	99%	96%	101%
2. Intermediate occupations	101%	102%	104%	99%	103%
3. Routine and manual occupations	99%	100%	101%	102%	99%
*Never worked and long-term unemployed	103%	99%	104%	100%	100%
**Male**	**20-29**	**30-39**	**40-49**	**50-59**	**60-69**
1. Higher managerial, administrative and professional occupations	103%	98%	105%	100%	98%
2. Intermediate occupations	101%	96%	101%	100%	103%
3. Routine and manual occupations	100%	99%	101%	99%	100%
*Never worked and long-term unemployed	101%	99%	100%	100%	101%

Table S4: Calculation of average fuel costs per mile from the CO2 emissions data.

	CO2 (kg CO2/mile) (5)	Kg CO2/litre) (6)	Litre/mile	Fuel costs (p/litre)	Average fuel cost (£/mile) (7)	% of registered cars (8)	Average cost per mile (£/mile)
Petrol	0.32	2.31	0.14	142	0.20	70%	0.14
Diesel	0.30	2.66	0.11	148	0.17	30%	0.05
Sum	-	-	-	-	-	100%	**0.19**

Table S5: Number of deaths averted in different scenarios, including uncertainty. See Figure 2 for illustration. CI = Credible Interval.

**Scenario**	**2.5% CI**	**Mean**	**97.5% CI**
A	47	75	113
B	570	811	1,075
C	1,664	2,284	3,093
D	3,365	4,904	7,165
E	4,924	7,648	11,657
F	1,444	3,255	4,932
G	1,296	5,063	8,867
H	-198	5,894	11,734
I	-1,755	6,139	13,991
J	-2,430	6,187	14,836
K	-749	6,158	12,994

Figure S2: Mean increase in cycling (min/week) by gender and scenario for scenarios A-E for those that change from car to bicycle.

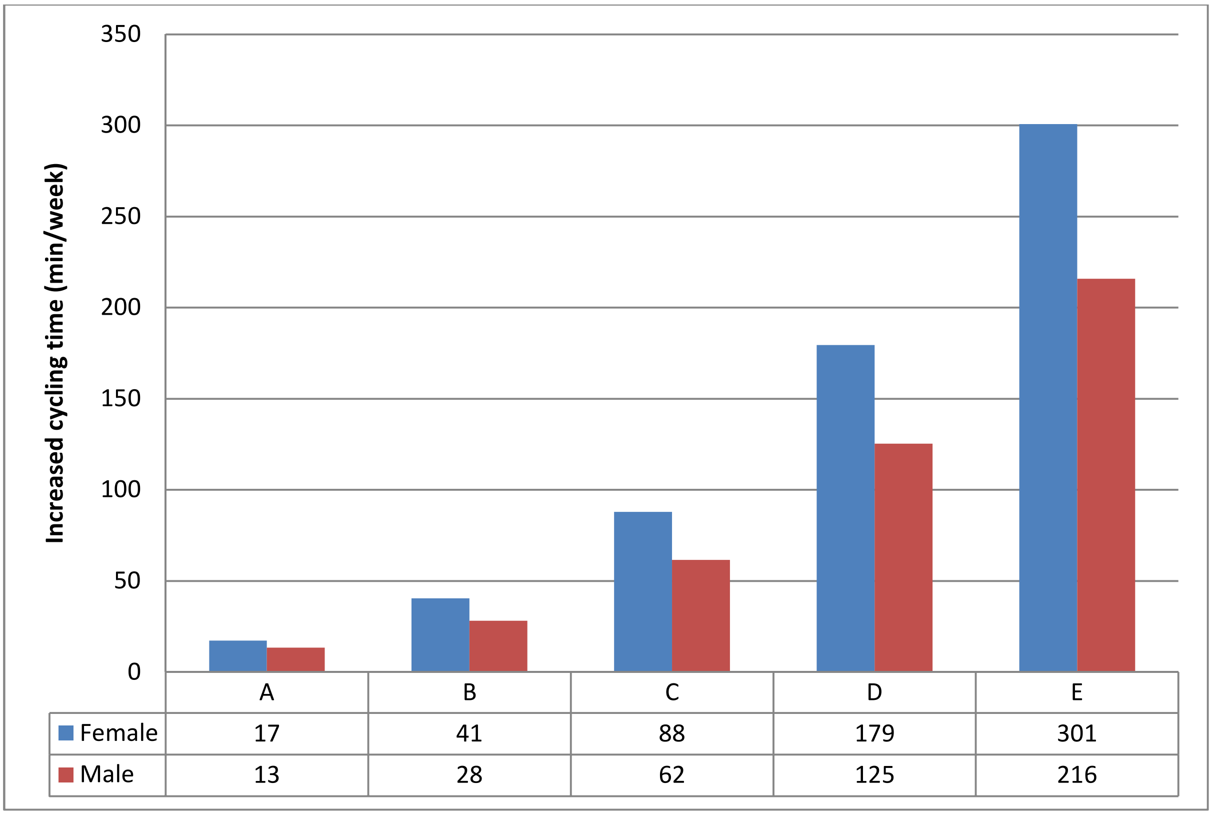

Figure S3: Percentage of people doing at least 150 and 300 minutes physical activity in different scenarios. Percentage was calculated by comparing the mMETs of average people in different scenarios to target of 8.25 and 17.5 mMETHs per week, for 150 and 300 minutes, respectively.

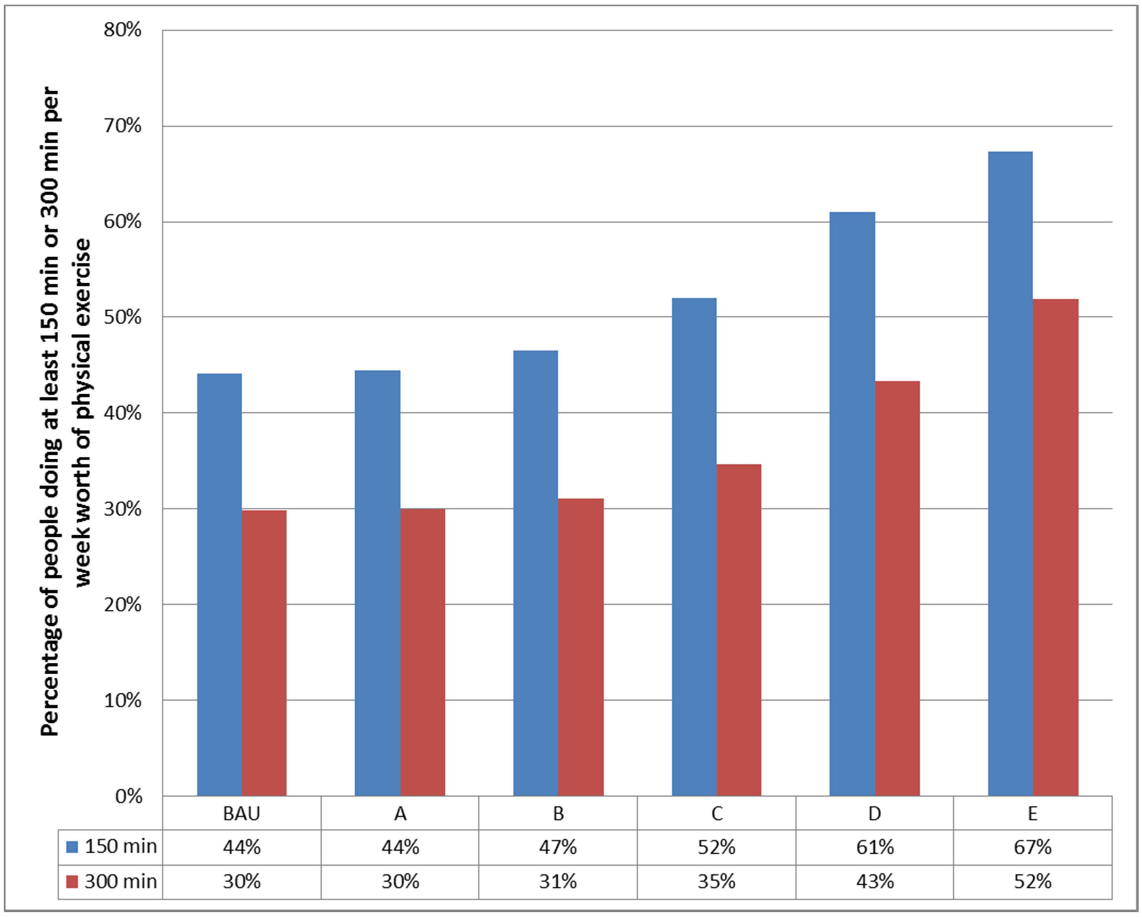

	BAU	A	B	C	D	E
150 min	44%	44%	47%	52%	61%	67%
300 min	30%	30%	31%	35%	43%	52%

Figure S4: Average percentage change in all-cause mortality by scenario and NS-SEC.

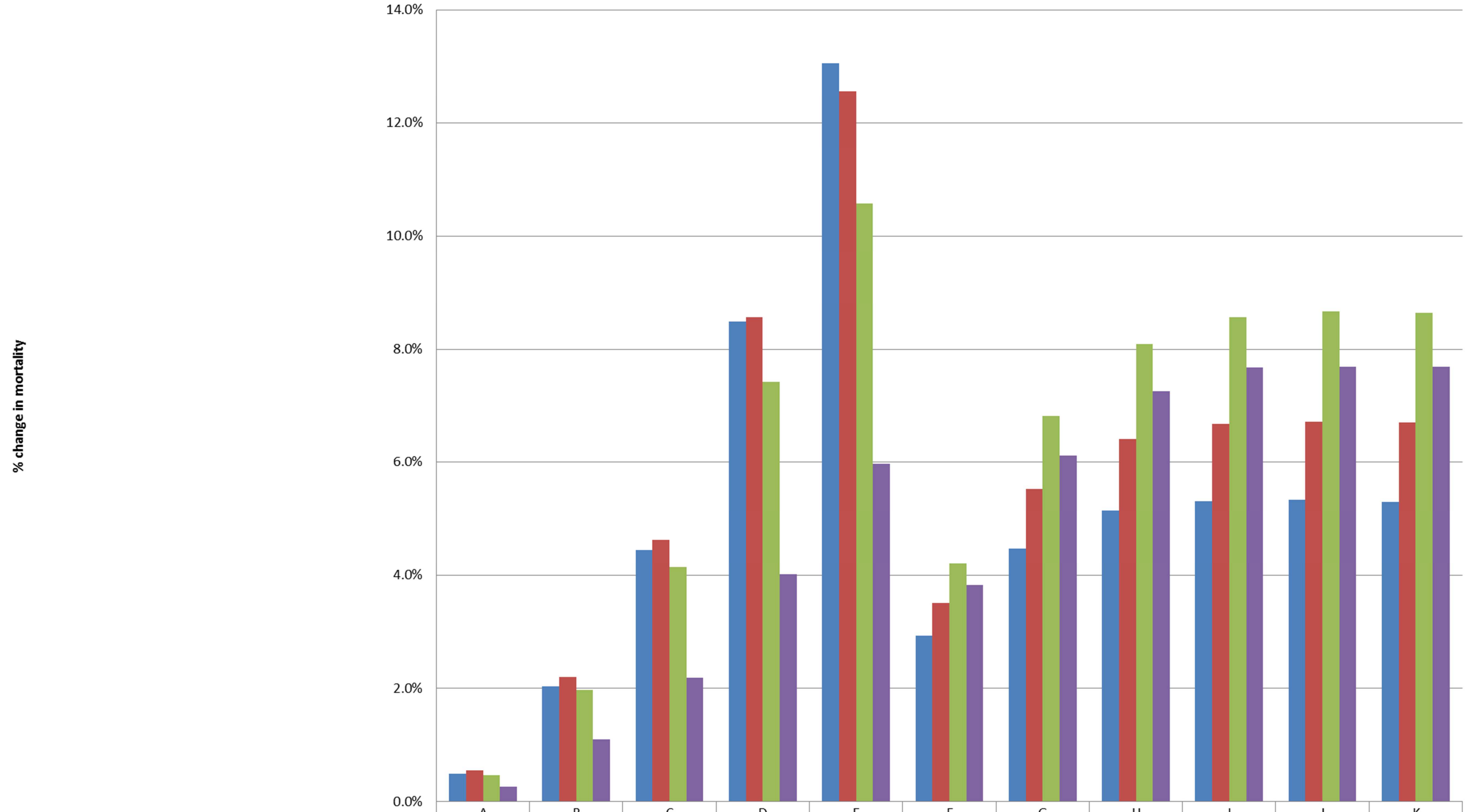

	A	B	C	D	E	F	G	H	I	J	K
1. Higher managerial, administrative and professional occupations	0.5%	2.0%	4.4%	8.5%	13.1%	2.9%	4.5%	5.1%	5.3%	5.3%	5%
2. Intermediate occupations	0.6%	2.2%	4.6%	8.6%	12.6%	3.5%	5.5%	6.4%	6.7%	6.7%	7%
3. Routine and manual occupations	0.5%	2.0%	4.1%	7.4%	10.6%	4.2%	6.8%	8.1%	8.6%	8.7%	9%
*Never worked and long-term unemployed	0.3%	1.1%	2.2%	4.0%	6.0%	3.8%	6.1%	7.3%	7.7%	7.7%	8%

Figure S5: Average reduction in premature mortality by scenario and NS-SEC.

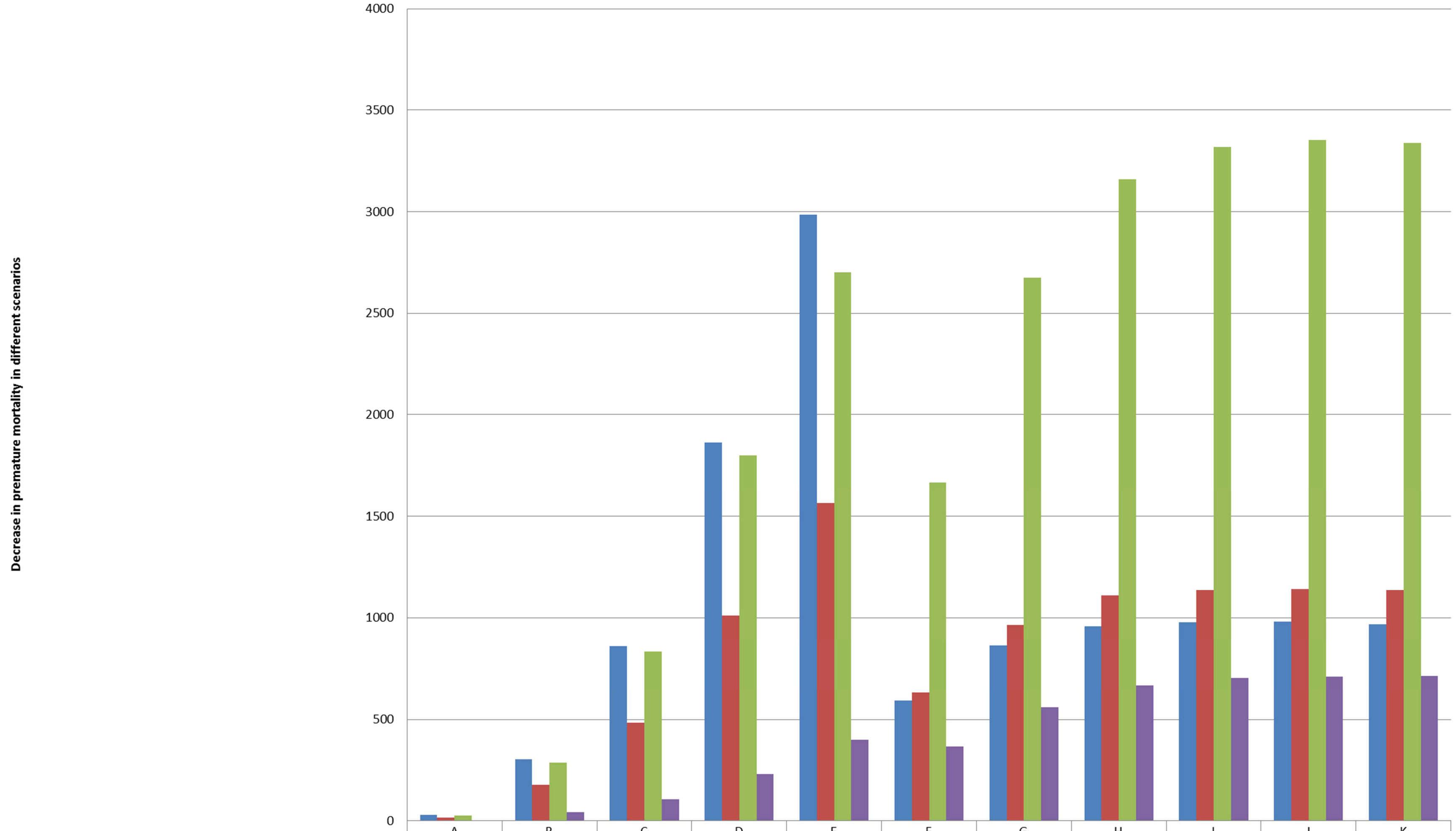

	A	B	C	D	E	F	G	H	I	J	K
1. Higher managerial, administrative and professional occupations	29	304	860	1862	2985	594	864	959	978	980	970
2. Intermediate occupations	16	179	485	1010	1564	632	967	1109	1138	1142	1137
3. Routine and manual occupations	26	287	833	1800	2703	1666	2675	3159	3319	3353	3337
*Never worked and long-term unemployed	4	41	106	231	397	364	558	667	704	712	714

Table S6: Changes in per person costs for diet (A) and physical activity (B) scenarios and comparison of the changes to background transport and food & non-alcoholic drinks costs. Background costs are based on Family Spending 2012 (9).

A

	Household expenditure per week			Expenditure per person		Increase in food & non-alcoholic drinks costs per scenario						Percentage increase in costs					
**NS-SEC group**	**Food & non-alcoholic drinks**	**Fruits and vegetables**	**Average number of persons per household (average)**	**Food & non-alcoholic drinks**	**Fruits and vegetables**	**F**	**G**	**H**	**I**	**J**	**K**	**F**	**G**	**H**	**I**	**J**	**K**
1. Higher managerial, administrative and professional occupations	£ 66.03		2.7	£ 24.09		£ 1.57	£ 3.14	£ 4.70	£ 6.27	£ 7.84	£ 1.99	107%	113%	120%	126%	133%	108%
2. Intermediate	£ 58.57		2.6	£ 22.17		£ 1.57	£ 3.14	£ 4.70	£ 6.27	£ 7.84	£ 2.50	107%	114%	121%	128%	135%	111%
3. Routine and manual occupations	£ 55.27		2.7	£ 20.25		£ 1.57	£ 3.14	£ 4.70	£ 6.27	£ 7.84	£ 2.98	108%	115%	123%	131%	139%	115%
*Never worked and long-term unemployed	£ 45.24		1.9	£ 24.06		£ 1.57	£ 3.14	£ 4.70	£ 6.27	£ 7.84	£ 2.62	107%	113%	120%	126%	133%	111%
**Average population**	**£ 54.80**	**£ 8.10**	**2.4**	**£ 22.83**	**£ 3.38**	**£ 1.57**	**£ 3.14**	**£ 4.70**	**£ 6.27**	**£ 7.84**	**£ 2.49**	**107%**	**114%**	**121%**	**127%**	**134%**	**111%**

B

						Increase in transport costs per scenario					Percentage change in costs				
**NS-SEC group**	**Transport: All**	**Transport: Operation of personal transport**	**Average number of persons per household (average)**	**Transport: All**	**Transport: Operation of personal transport**	**A**	**B**	**C**	**D**	**E**	**A**	**B**	**C**	**D**	**E**
1. Higher managerial, administrative and professional occupations	£ 108.43		2.7	£ 39.57		-£ 0.07	-£ 0.38	-£ 1.10	-£ 2.71	-£ 5.05	100%	99%	97%	93%	87%
2. Intermediate	£ 72.59		2.6	£ 27.47		-£ 0.07	-£ 0.37	-£ 1.03	-£ 2.53	-£ 4.54	100%	99%	96%	91%	83%
3. Routine and manual occupations	£ 58.70		2.7	£ 21.51		-£ 0.05	-£ 0.29	-£ 0.84	-£ 2.04	-£ 3.70	100%	99%	96%	91%	83%
*Never worked and long-term unemployed	£ 35.72		1.9	£ 19.00		-£ 0.02	-£ 0.16	-£ 0.44	-£ 1.07	-£ 1.88	100%	99%	98%	94%	90%
**Average population**	**£ 65.70**	**£ 36.40**	**2.4**	**£ 27.38**	**£ 15.17**	**-£ 0.06**	**-£ 0.33**	**-£ 0.93**	**-£ 2.28**	**-£ 4.17**	**100%**	**99%**	**97%**	**92%**	**85%**

Figure S6: Percentage of people changing from car to bicycle in scenarios A-E, divided by different NS-SEC groups. In all scenarios larger fraction of people in highest SES group increased cycling.

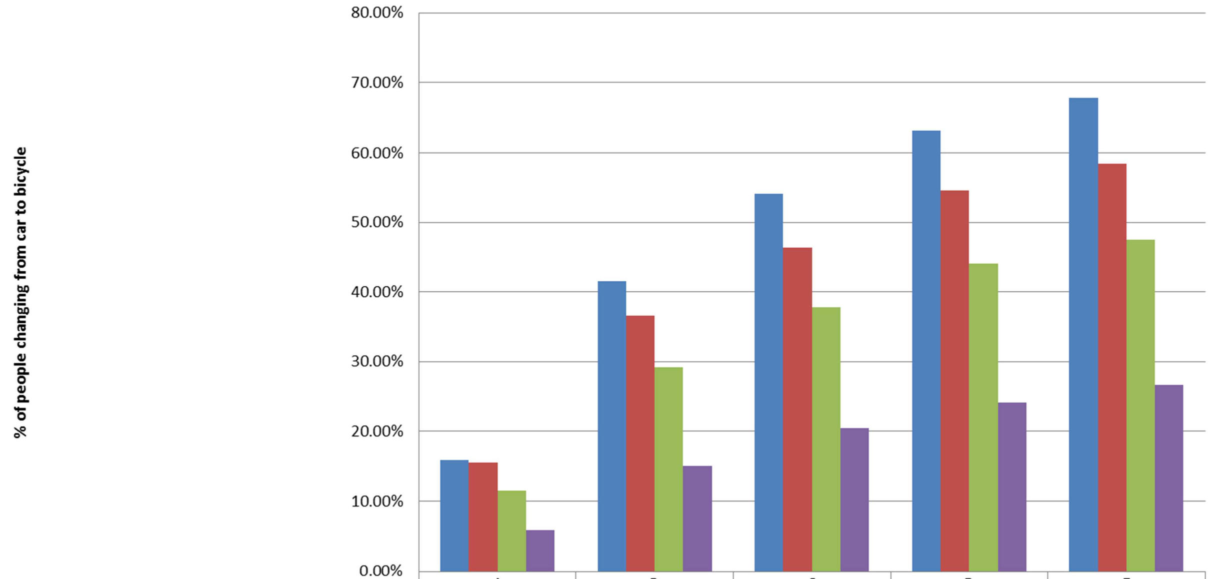

	A	B	C	D	E
1. Higher managerial, administrative and professional occupations	15.87%	41.49%	54.07%	63.16%	67.88%
2. Intermediate occupations	15.55%	36.63%	46.32%	54.49%	58.39%
3. Routine and manual occupations	11.46%	29.16%	37.75%	43.99%	47.49%
*Never worked and long-term unemployed	5.93%	15.06%	20.47%	24.15%	26.63%

Comparison to previous studies

Physical activity and health*:* Rojas-Rueda et al. (10) estimated the health benefits and risks of replacing 40% of car trips inside the Barcelona, Spain, with cycling. The average distance of the trip was assumed to be 3.1 km and the mortality benefits were estimated for 16–64 year olds. The resulting physical activity benefits were 67 avoided death per year for the population of 1.6 million. If we scale this to our study population and assume that 100% of the inside city trips would be shifted to cycling, the result would be approximately 3600 avoided death per year. This result is similar to scenarios C and D in our study (Figure 2).

New Zealand study estimated health benefits and GHG emission changes of shifting less than 7 km long car trips with cycling (11). They found out that if 30% of the trips would be replaced with cycling, the health benefits of physical activity would be approximately 700 deaths per year. If we scale their results for our study population, the comparable number of deaths avoided would be 31 000 per year. This is five times more than scenario D in our study (Figure 2).

Diet: Scarborough et al. (12) modelled health benefits of eating one apple per day more through changes in cardiovascular mortality by using the PRIME model. One apple was assumed to weight 100 g (1.25 portions in our study). The mean annual average cardiovascular deaths avoided were 8500. In our study scenario F examined health benefits of eating one portion of F&V more per day with the impact of 3 255 avoided deaths for the 20-69 year old. Due to population age differences direct comparison of studies is not possible but the magnitude of the impact is similar, with our study estimating approximately half of the health benefits.

Study in Wirral, UK, examined several lifestyle related scenarios for a population of 312 000 (13). One of the modelled scenarios assumed that study population would eat at least 600g F&V per day. This scenario was estimated to prevent 164 deaths per year. If scaled to our study population of 34 million, this would represent 18 000 avoidable deaths per year; a result about three times higher than 6 158 avoidable deaths in scenario K in this study.

References

1. Office for National Statistics. Standard Occupational Classification 2010. Volume 3. The National Statistics Socio-economic Classification : (Rebased on the SOC2010) User Manual [Internet]. 2010. Available from: http://www.ons.gov.uk/ons/guide-method/classifications/current-standard-classifications/soc2010/soc2010-volume-3-ns-sec--rebased-on-soc2010--user-manual/index.html

2. Department for Transport. National Travel Survey, 2002-2012 [Internet]. 8th Editio. Colchester, Essex: UK Data Archive; 2014. Available from: http://discover.ukdataservice.ac.uk/catalogue/?sn=5340

3. Ainsworth BE, Haskell WL, Herrmann SD, Meckes N, Bassett DR, Tudor-Locke C, et al. 2011 Compendium of Physical Activities: a second update of codes and MET values. Med Sci Sports Exerc [Internet]. 2011 Aug [cited 2014 Nov 21];43(8):1575–81. Available from: http://www.ncbi.nlm.nih.gov/pubmed/21681120

4. NatCen Social Research UCLD of E and PH. Health Survey for England, 2012 [Internet]. 2014. Available from: https://discover.ukdataservice.ac.uk/Catalogue/?sn=7480&type=Data catalogue

5. Department for Environment Food & Rural Affairs (DEFRA). Greenhouse Gas Conversion Factor Repository [Internet]. 2015. Available from: http://www.ukconversionfactorscarbonsmart.co.uk/

6. Iain MacLeay, Harris K, Annut A, Authors and C. Digest of United Kingdom Energy Statistics (DUKES) 2013 [Internet]. 2013. 1-268 p. Available from: https://www.gov.uk/government/publications/digest-of-united-kingdom-energy-statistics-dukes-2013-printed-version-excluding-cover-pages

7. Department for Energy and Climate Change (DECC). Petrol and diesel prices and duties per litre in April: United Kingdom from 20001 [Internet]. 2015. Available from: https://www.gov.uk/government/uploads/system/uploads/attachment_data/file/48915/env0105.xls

8. Department for Environment F and RA (DEFRA). 2012 Guidelines to Defra / DECC's GHG. Conversion Factors for Company Reporting: Methodology Paper for Emission Factors [Internet]. Methodology Paper for Emission Factors. 2012. Available from: http://tinyurl.com/p46qukp

9. Daniel E, Keyse L, Skilton L, Karim S, Mahoney T. Family Spending, 2012 Edition [Internet]. Horsfield G, editor. 2012. Available from: http://webarchive.nationalarchives.gov.uk/20160105160709/http://ons.gov.uk/ons/rel/family-spending/family-spending/family-spending-2012-edition/index.html

10. Rojas-Rueda D, de Nazelle A, Teixid?? O, Nieuwenhuijsen MJ. Replacing car trips by increasing bike and public transport in the greater Barcelona metropolitan area: A health impact assessment study. Environ Int [Internet]. Elsevier Ltd; 2012;49:100–9. Available from: http://dx.doi.org/10.1016/j.envint.2012.08.009

11. Lindsay G, Macmillan A, Woodward A. Moving urban trips from cars to bicycles: Impact on health and emissions. Aust N Z J Public Health. 2011;35(1):54–60.

12. Scarborough P, Harrington RA, Mizdrak A, Zhou LM, Doherty A. The Preventable Risk Integrated ModEl and Its Use to Estimate the Health Impact of Public Health Policy Scenarios. Scientifica (Cairo) [Internet]. 2014;2014:1–21. Available from: http://www.hindawi.com/journals/scientifica/2014/748750/

13. Möller H, Dherani M, Harwood C, Kinsella T, Pope D. Health planning for the future: Comparative risk assessment of five major lifestyle risk factors: Evidence from the Wirral, UK. J Public Heal (United Kingdom). 2012;34(3):430–7.
